# Positive Relational Management for Healthy Organizations: Psychometric Properties of a New Scale for Prevention for Workers

**DOI:** 10.3389/fpsyg.2016.01523

**Published:** 2016-10-13

**Authors:** Annamaria Di Fabio

**Affiliations:** Department of Education and Psychology (Psychology Section), University of FlorenceFlorence, Italy

**Keywords:** positive relational management, psychometric properties, positive relational management scale, positive psychology, inclusive psychology of working, inclusive relational perspective, relational theory of working

## Abstract

This contribution aims at evaluating the psychometric properties of the Positive Relational Management Scale (PRMS) in a sample of 251 Italian workers. The dimensionality, reliability, and concurrent validity of the scale were investigated. Confirmatory factor analysis supported a correlated three-dimensional version of the scale, comprising Respect, Caring, and Connectedness. Latent correlations among the dimensions were moderate-to-strong (0.44–0.57), but suggestive of the multidimensionality of the scores. In addition, good internal consistency was confirmed. The concurrent validity is good as the Pearson’s correlations between PRMS and measure for social support, life satisfaction, life meaningfulness, and flourishing range from 0.39 to 0.52. The results indicate that the PRMS is a valid instrument for measuring positive relational management at work in the Italian context within a positive preventive perspective.

## Introduction

### The Importance of Relationships

Interpersonal relationships and relational experiences are of primary importance for work success (Blustein et al., 1995; Schultheiss et al., 2001; Kenny et al., 2003; Blustein, 2006). In the field of work and organizational psychology, Blustein (2006) in his inclusive psychology of working states that relationships are an important need of people and that working can be coupled with three basic needs: the need for survival and power, the need for social relationships, and the need for self-determination. Within a relational perspective Blustein (2011) later maintains that the work is an inherently relational act: relationships influence and shape every decision, experience, and interaction of individuals in the world of work (Blustein, 2011; Di Fabio, 2014a). Relationships can thus be considered fundamental resources for workers, highlighting the importance of creating optimal conditions to support adaptive relationships in organizational contexts from a positive psychology perspective (Seligman and Csikszentmihalyi, 2000; Seligman, 2002) in terms of flourishing relationships (Di Fabio, 2014a; Snyder et al., 2014). The focus should therefore be on helping people build lives through work and relationships (Blustein, 2011; Richardson, 2012). This calls for a positive organizational psychology approach that underlines the importance of developing positive and supportive relationships in the workplace (Snyder et al., 2014; Di Fabio, 2015a,b; Di Fabio and Gori, 2016b).

### Healthy Organization and Healthy Business

The positive organizational psychology framework (Snyder et al., 2014; Di Fabio, 2015b) also involves the concept of the healthy organization (De Smet et al., 2007) implying the relevance of the health and well-being of workers for business and organizational health (Danna and Griffin, 1999; Di Fabio et al., 2014; Grawitch and Ballard, 2016). The focus on the concept of health in organizations is in line with the definition of health by World Health Organization [WHO] (1998). Indeed health is not merely the absence of disease: “it is a state of complete physical, mental, spiritual and social well-being and not merely the absence of disease or infirmity” (World Health Organization [WHO], 1998). Thus healthy people in organizations are flourishing and resilient workers on the one side; on the other side positive work environment focuses on workers’ health, well-being and joint performance for healthy organizations (Di Fabio and Busoni, 2007; Di Fabio, 2014a; Snyder et al., 2014; Di Fabio and Gori, 2016b; Di Fabio and Kenny, 2016a). Substantially healthy organizations promote healthy businesses (Grawitch and Ballard, 2016) by taking into account not only performance but at the same time workers’ as well as organizations’ well-being (Di Fabio and Kenny, 2016a; Di Fabio and Maree, 2016). The concept of healthy organization (De Smet et al., 2007) highlights the importance of health and performance of individuals for organizational success and business, effectiveness and functioning (Danna and Griffin, 1999) and underlines the sound relationships between workers and organizational well-being. A healthy organization is characterized by both good profit and health business (Grawitch and Ballard, 2016) and the well-being of workers.

In order to promote healthy organizations and healthy businesses, the concepts of gainful employment and life (Di Fabio, 2014a, 2015b) and new ways of conceptualizing organizational relationality require urgent attention (Di Fabio, 2015b).

### Relationships and Well-Being in Organizations

In the current unstable and insecure world of work, the well-being of individuals in organizations is under threat (Di Fabio, 2014a,b, 2015b; Di Fabio et al., 2016a). Because relationships are a key factor in people’s well-being (Gallagher and Vella-Brodrick, 2008; Suldo et al., 2009; Ferguson and Goodwin, 2010; Blustein, 2011; Kalpana Rani, 2016), fostering positive relationships can promote well-being in the workplace (Di Fabio, 2014a, 2015b; Snyder et al., 2014; Di Fabio and Gori, 2016b). The attention to a condition of well-being can also improve the opportunities of workers to adapt themselves to changes and uncertainty of the postmodern world of work (Di Fabio and Kenny, 2016a).

Particularly from a positive preventive perspective (Hage et al., 2007; Kenny and Hage, 2009; Di Fabio and Kenny, 2011, 2012, 2015, 2016b; Kenny et al., 2014; Di Fabio et al., 2016a), it is crucial to anticipate critical situations in order to intervene early as well as identify promptly resources that can promote people’s well-being (Di Fabio and Bucci, 2015; Di Fabio and Palazzeschi, 2015a, 2016) and optimal functioning (Di Fabio et al., 2016a). In line with American Psychological Guidelines (Hage et al., 2007) early interventions are more effective when efforts to increase resources and competencies (World Medical Association [WMA], 2013; Boyatzis et al., 2002, 2015; Di Fabio, 2006; Boyatzis and Saatcioglu, 2008; Di Fabio and Bernaud, 2008; Boyatzis, 2009) are combined with efforts to decrease risks (Di Fabio and Kenny, 2011; Di Fabio, 2014a; Di Fabio and Saklofske, 2014a,b; Di Fabio et al., 2014). The availability of instruments able to detect relational resources in different contexts and in particular in the workplace, is important for early interventions to promote well-being focusing on individual and organizational strengths (Di Fabio, 2014a). A positive workplace relational environment needs to be created to enable workers to increase their personal resources to face the ever-changing word of work, to accept the inevitability of change (Di Fabio and Gori, 2016a), and to promote their well-being (Di Fabio, 2014a; Snyder et al., 2014; Di Fabio and Gori, 2016a; Di Fabio and Kenny, 2016a).

In a positive preventive perspective (Di Fabio and Kenny, 2016a) the focus of interventions aiming at promoting well-being in order to gain balance between oneself and other individuals and context, asks for the detection and promotion of the following aspects: respects for me and others, take care of me and of others, the connection and strong relationships with me and with others (Di Fabio, 2014a, 2015b). Positive preventive perspective (Hage et al., 2007; Kenny and Hage, 2009; Di Fabio et al., 2016a) highlights the need for individuals to enhance and manage positive relationships (Di Fabio and Palazzeschi, 2008, 2012a,b; Di Fabio et al., 2012, 2013b, 2015; Di Fabio, 2014a,b, 2015b) and to improve respectivity (Maree, 2012) and relationality (Blustein, 2011). For individuals taking care of self and others is important for flourishing in the construction of new chapters of their personal and professional lives (Di Fabio, 2014a).

### Positive Self and Relational Management

Positive relational management is central in the Positive Self and Relational Management (PS&RM, Di Fabio and Kenny, 2016a) model. PS&RM (Di Fabio and Kenny, 2016a) draw upon the *career and life management through self and relational management* (Di Fabio, 2014a; Di Fabio and Kenny, 2016a) integrating work and relationship in a dialectical manner across the lifespan (Di Fabio, 2014a), and strengthening many aspects of the self in a relational context. PS&RM (Di Fabio and Kenny, 2016a) defines lifelong development as “the development of individuals’ strengths, potentials and varied talents from a lifespan perspective and the positive dialectic of the self in relationship” (Guichard, 2004, 2005, 2009, 2013; Blustein, 2011; Savickas, 2011; Di Fabio, 2014a, 2015b). In PS&RM (Di Fabio and Kenny, 2016a) the construct of positive lifelong relational management is fundamental, including relational and social skills, such as emotional intelligence and social support, and competencies useful to manage the current challenges. In this model the construct of Positive relational management emerges, relying on the ideas of respect and caring for the self and others and the relationships between people (Blustein, 2006; Di Fabio and Maree, 2012, 2016; Maree, 2013; Di Fabio, 2015b).

Moving from the importance of the concepts of relationality, respectivity and caring toward oneself and others in the relationships (Blustein, 2006; Di Fabio and Maree, 2012, 2016; Maree, 2013; Di Fabio, 2015b; Di Fabio and Bucci, 2016), central in the positive relational management construct, a new instrument was developed, the Positive Relational Management Scale (PRMS; Di Fabio, 2015b).

The scale consists of 12 items, four for each dimension: *Respect* (my respect for others, the respect of others for me, my respect for myself), *Caring* (my care for others, the care of others for me, my care for myself), and *Connectedness* (my connectedness with family members, with friends, with significant others).

These aspects are not comprised by other construct and relative measure, such as perceived social support received by others (Zimet et al., 1988) or flourishing (Diener et al., 2010), a process of growth in which social relationships are one of the main aspects for individual.

A pilot study (Di Fabio, 2015b) with Italian university students empirically confirmed the theoretical three-dimensional structure of the scale. In this study an exploratory factor analysis (EFA) was firstly conducted and then a confirmatory factor analysis (CFA) was used to analyze the three-dimensional structure of the PRMS. The goodness-of-fit indices were good for the ratio of the chi-square to degrees of freedom (χ/gdl = 1.64) acceptable for the Comparative fit Index (CFI = 0.92) and the Non-Normed Fit Index (NNFI = 0.94), and acceptable for the Root Mean Square Error of Approximation (RMSEA = 0.07) (Browne and Cudeck, 1993; Marsh et al., 2004). The scale presented also a good reliability (Cronbach’s alpha coefficient are 0.79 for Caring, 0.80 for Connection, 0.81 for Respect, and 0.84 for PRMS total).

Because positive relational management is a resource in university students, in line with previous studies (Di Fabio, 2015b; Di Fabio and Kenny, 2016a) it is expected as a resource also for relational productive and positive adaptation in the workplace (Di Fabio and Kenny, 2016a). In Italian university students associations between positive relational management and social support emerged (Di Fabio, 2015b; Di Fabio and Kenny, 2016a), showing the importance to nurture sound relationships (Di Fabio et al., 2013a; Kozan et al., 2014). Some studies reported also the positive association between positive relational management and hedonic well-being in terms of life satisfaction (Di Fabio and Kenny, 2016a), and eudaimonic well-being in terms of life meaningfulness and flourishing (Di Fabio and Kenny, 2016a), indicating that positive relationships can promote individual well-being (Arnoux-Nicolas et al., 2016). The value of positive relational management was also underlined in an organizational context regarding workplace relational civility (Di Fabio and Gori, 2016b).

Against this theoretical background, and on the basis of the good results of the pilot study (Di Fabio, 2015b) with regard to Italian university students, the aim of the present study was to evaluate the psychometric properties of the PRMS by Di Fabio (2015b) also in Italian workers.

## Materials and Methods

### Participants

Two hundred and fifty-one workers of different public and private organizations in central Italy participated in the study. Regarding gender, 158 were men (62.95%) and 93 were women (37.05%), with a mean age of 38.75 (*DS* = 10.02). Regarding occupations, 143 (57.97%) were white collars and 108 (42.03%) were blue collars.

### Measures

#### Positive Relational Management Scale (PRMS) for Workers

The PRMS is a measure consisting of 12 items on a five-point Likert scale ranging from 1 (*strongly disagree*) to 5 (*strongly agree*). The PRMS detects three dimensions (Respect, Caring, Connectedness) and also has a total score (e.g., Respect: “I keep a balance between respect toward others and toward myself”; Caring: “I often take care of others”; Connectedness: “I have good relationships with my family”). The Cronbach’s alpha coefficients in the previous study with Italian university students (Di Fabio, 2015b) were the following: for Respect 0.81; for Caring 0.79; for Connection 0.80. Regarding the concurrent validity, the correlations between the PRMS and perceived social support were 0.36 for Respect, 0.38 for Caring, 0.42 for Connectedness, and 0.41 for the total (Di Fabio, 2015b).

The psychometric properties of the PRMS for workers were analyzed in the present study. The items of the PRMS are shown in the **Appendix** at the end of this article.

#### Multidimensional Scale for Perceived Social Support (MSPSS)

The Multidimensional Scale for Perceived Social Support (MSPSS; Zimet et al., 1988) in the Italian version by Di Fabio and Palazzeschi (2015a) was used to evaluate perceived social support. The scale consists of 12 items with response options on a seven-point Likert scale ranging from 1 = *strongly disagree* to 7 = *strongly agree*. The MSPSS detects perceived support from family members (example of item: “My family works very hard to help me”), friends (example of item: “I can speak about my problems with my friends”), significant others (example of item: “When I need someone, there is always a special person who stands by me”). Cronbach’s alpha coefficients were 0.92 for the Family dimension, 0.90 for the Friends dimension, 0.93 for the Significant others dimension, and 0.91 for the total score. Regarding the concurrent validity, the correlations between the MSPSS and the Italian version of SWLS (Di Fabio and Gori, 2015) were 0.49 for Perceived support from family; 0.42 for Perceived support from friends; 0.39 for Significant others dimension; and 0.51 for the total score.

#### Satisfaction With Life Scale (SWLS)

Life satisfaction was assessed using the Italian version (Di Fabio and Gori, 2015) of the Satisfaction With Life Scale (SWLS, Diener et al., 1985). The questionnaire consists of five items, which are rated using a seven-point Likert scale that ranges from 1 = *Strongly disagree* to 7 = *Strongly agree*. The Cronbach’s alpha coefficient reported by Di Fabio and Gori (2015) was 0.85. Regarding the concurrent validity, the correlations between the SWLS were 0.57 with the Italian version of Rosenberg Self-Esteem Scale (Prezza et al., 1997); 0.51 with the Italian version of MSPSS (Di Fabio and Palazzeschi, 2015a); and 0.65 with the Italian version of TEIQue (Di Fabio et al., 2016b).

#### Meaningful Life Measure (MLM)

The Meaningful Life Measure (MLM, Morgan and Farsides, 2009) in the Italian version by Di Fabio (2014d) was used to evaluate meaning in life. The questionnaire consists of 23 items with response options on a seven-point Likert scale ranging from 1 = *strongly disagree* to 7 = *strongly agree*. The MLM detects five dimensions: Exciting life, Accomplished life, Principled life, Purposeful life, Valued life. Cronbach’s alpha coefficient for the total score was 0.82. Regarding the concurrent validity, the correlations between the MLM were 0.58 with the Italian version of Meaning in Life Questionnaire (MLQ, Di Fabio, 2014e); 0.43 with the Italian version of SWLS (Di Fabio and Gori, 2015); 0.49 with Positive Affect and -0.20 with Negative Affect of the Italian version of Positive and Negative Affect Schedule (PANAS, Terraciano et al., 2003).

#### Flourishing Scale

The Flourishing Scale (Diener et al., 2010) in the Italian version by Di Fabio (2016a) was used to evaluate flourishing. This measure consists of eight items with response options on a seven-point Likert scale ranging from 1 = *strongly disagree* to 7 = *strongly agree*. Examples of items are “My social relationships are supportive and rewarding,” “I lead a purposeful and meaningful life,” “I am optimistic about my future.” Cronbach’s alpha coefficient was 0.88. Regarding the concurrent validity, the correlations between the Flourishing Scale were 0.69 with the Italian version of MLM (Di Fabio, 2014d); 0.48 with the Italian version of the Authenticity Scale (Di Fabio, 2014c); 0.48 with the Italian version of SWLS (Di Fabio and Gori, 2015); 0.54 with Positive Affect and -0.33 with Negative Affect of the Italian version of Positive and Negative Affect Schedule (PANAS, Terraciano et al., 2003).

### Procedure

The instruments/questionnaires were administered to groups of respondents by trained psychologists. The administration sequence was alternated to counter the possible effects of a set presentation of the instruments. The instruments were administered according to the requirements of privacy and informed consent of Italian law (Law Decree DL-196/2003). Regarding ethical standards for research, the study adhered to the latest version of the Declaration of Helsinki revised in Fortaleza (World Medical Association [WMA], 2013). They were followed and approved by the Department of Education and Psychology of the University of Florence (Italy).

### Data Analysis

Confirmatory factor analysis, conducted through the maximum likelihood estimation using Mplus version 7 (Muthén and Muthén, 1998–2015), was used to analyze the factor structure of the PRMS for workers, and multiple goodness-of-fit indices were used to assess statistically the closeness of the hypothetical model to the empirical data. The reliance on fit indices, as opposed to statistical tests of fit like the chi-square, to determine model fit emerges from limitations with the chi-square. Standard goodness-of-fit indexes were used including the chi-square (χ2), the Tucker-Lewis Index (TLI), the Comparative Fit Index (CFI), the Standardized Root Mean Square Residual (SRMR), and the Root Mean Square Error of Approximation (RMSEA). Values greater than 0.90 and 0.95 of CFI and TLI are indicative of acceptable and excellent fit, respectively; and SRMR and RMSEA values lower than 0.05 and 0.08 are suggestive of close and reasonable fit, respectively (Browne and Cudeck, 1993; Marsh et al., 2004), and ideally equal to or less than 0.05, are indices of good fit (Steiger, 1990; Schermelleh-Engel et al., 2003; Giannini et al., 2011). The reliability of the PRMS was confirmed through Cronbach’s alpha coefficient and corrected item-total correlations. Pearson’s correlations between the PRMS and the MSPSS, SWLS, MLM, and FS were used to verify several aspects of concurrent validity.

## Results

**FIGURE 1:**
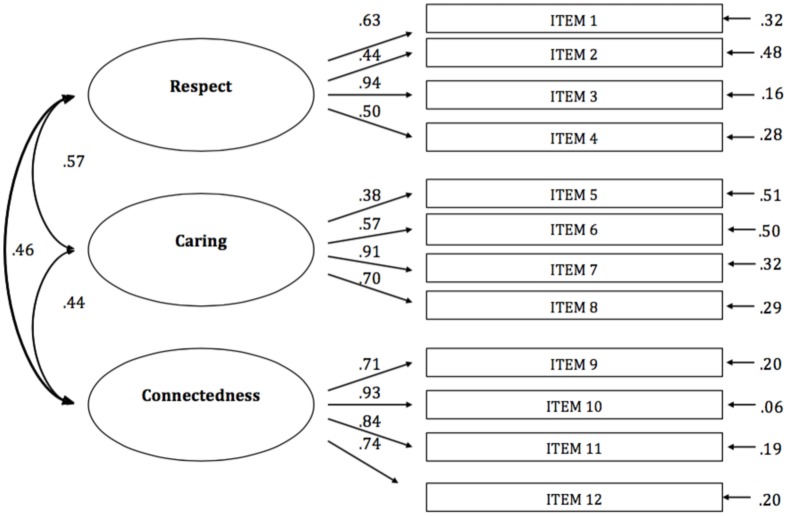
**Confirmatory factor analysis (CFA) of the theoretical three-factor structure of the Positive Relational Management Scale (PRMS)**.

A CFA was carried out to confirm the structure of the PRMS for workers (**Figure 1**).

The goodness of fit indices are acceptable for the three-dimensional structure of the PRSM. The chi-square, degree of freedom, and probability values for the three-dimensional model are χ^2^(51) = 135.42 (*p* < 0.001). The other goodness-of-fit indices showed acceptable and reasonable values (CFI = 0.93; TLI = 0.91; SRMR = 0.07; RMSEA = 0.05).

The goodness of fit indices of the bi-dimensional model (TLI = 0.83; CFI = 0.87; RMSEA = 0.11; SRMR = 0.06; BIC = 5975.927) and of the uni-dimensional model tested (TLI = 0.60; CFI = 0.67; RMSEA = 0.18; SRMR = 0.12) were lower than the three-factor model.

According to the literature (Raftery, 1995; Muthén and Muthén, 1998–2015) the three examined structures of the PRMS were compared, using the Mplus Bayesian Information Criterion (BIC) as a suitable statistical comparison. Comparison between BIC of two-factor model and three-factor model was 5975.927–5901.758 = 74.169; comparison between uni-dimensional model and three-factor model was 6220.609–5901.758 = 318.851. A value of comparison greater than 10 highlights a strong evidence that the model with the lower BIC fits better data, in this case the three-factor model of the PRMS.

Relating to the issue of gender invariance it was conducted MIMIC modeling analysis using Mplus 7. The results of the MIMIC model showed that gender had no significant effect regarding the dimensions of Caring and Connectedness; gender had a significant effect regarding the dimension of Respect.

Cronbach’s alpha coefficients for the three dimensions of the PRMS and for the total score were calculated to verify the internal consistency of the scale (**Table 1**).

**Table 1:** Cronbach’s alpha for the three dimensions and for the total score of the Positive Relational Management Scale for workers.

	Cronbach’s alpha
Respect	0.82
Caring	0.80
Connectedness	0.81
Positive Relational Management Scale total	0.85

*N = 251.*

The corrected item total correlations were also calculated (**Table 2**).

**Table 2:** Correlations item-total score of the Positive Relational Management Scale (PRMS).

	PRMS total score	PRMS respect	PRMS caring	PRMS connectedness
PRMS1	0.46	0.60		
PRMS2	0.55	0.66		
PRMS3	0.63	0.79		
PRMS4	0.65	0.68		
PRMS5	0.43		0.53	
PRMS6	0.49		0.62	
PRMS7	0.62		0.76	
PRMS8	0.65		0.72	
PRMS9	0.57			0.76
PRMS10	0.70			0.89
PRMS11	0.57			0.80
PRMS12	0.70			0.81

*N = 251.*

The correlations between the PRMS and the MSPSS, SWLS, MLM, and FL are shown in **Table 3**.

**Table 3:** Correlations of the Positive Relational Management Scale for workers with the MSPSS, SWLS, MLM, and FS.

	MSPSS	SWLS	MLM	FS
Respect	0.41**	0.49**	0.41**	0.43**
Caring	0.44**	0.52**	0.39**	0.41**
Connectedness	0.46**	0.50**	0.42**	0.47**
Total	0.45**	0.51**	0.43**	0.45**

N = 251. **p < 0.01. MSPSS, Multidimensional Scale for Perceived Social Support; SWLS, Satisfaction With Life Scale; MLM, Meaningful Life Measure; FS, Flourishing Scale.

## Discussion

The aim of the present study was to evaluate the psychometric properties of the PRMS (Di Fabio, 2015b) also for Italian workers. In line with the results of the pilot study carried out on Italian university students (Di Fabio, 2015b), the espoused three-factor model with its three dimensions was confirmed also in the present study with Italian workers, via CFA, as indicated by good indices of fit to the empirical data. Latent correlations of the three factors (Respect, Caring, Connectedness) indicate moderate relationships between the three factors (ranging from 0.44 to 0.57) supporting a three-factor structure of the PRMS. The reliability of the three dimensions and of the total score of the scale (verified through Cronbach’s alpha coefficient and corrected item-total correlations) was good.

Correlations between the PRSM and the measures used to verify aspects of concurrent validity showed good values, in line with previous studies. In particular, the positive correlation between the PRMS and the MSPSS, that emerged in the pilot study of the PRMS in Italian university students (Di Fabio, 2015b), indicated also the positive association between positive relational management at work and perceived social support, underlining the importance of developing positive and supportive relationships in different individual life contexts and particularly in the workplace, as reported in literature (Di Fabio, 2014a, 2015b; Snyder et al., 2014; Di Fabio and Kenny, 2016a). The quality of relationships at work can thus also enhance the quality of work produced and thereby promote healthy organizations (De Smet et al., 2007; Di Fabio, 2014a, 2015b; Di Fabio and Kenny, 2016a; Di Fabio and Maree, 2016).

Furthermore, the positive correlations between the PRMS and the SWLS, MLM (Di Fabio and Kenny, 2016a) and FS (Di Fabio and Gori, 2016b) indicated a positive association between positive relational management and both hedonic (SWLS) and eudaimonic well-being (MLM, FS) thus showing that positive relationships are a key factor in individual well-being in the workplace (Gallagher and Vella-Brodrick, 2008; Suldo et al., 2009; Ferguson and Goodwin, 2010; Blustein, 2011; Kalpana Rani, 2016). From a positive preventive point of view, positive relationships can promote well-being in the workplace (Di Fabio, 2014a, 2015b; Snyder et al., 2014; Di Fabio and Gori, 2016b), helping workers to positively live in the complexity of the current world of work (Di Fabio and Kenny, 2016a).

The results of the present study indicate that the PRMS can be considered a reliable and valid instrument for detecting the positive relational management construct at work. Nevertheless, some limitations of the study need mentioning. The psychometric properties of the PRMS for workers were investigated in a small group of Italian workers from different public and private organizations in central Italy who were not necessarily representative of all Italian workers. Future research should therefore include workers from other geographical areas in Italy and also from different types of organizations. It would also be interesting to compare the PRMS results for Italian workers with those for workers in other countries.

Furthermore, the use of Pearson’s correlations for verifying aspect of concurrent validity could be a limitation of the present study by virtue of their failure to controls for errors of measurement and partial out common method variance that may lead to upwardly biased relationships. In future studies it would be interesting to conduct these analyses in a latent framework, controlling for errors of measurements and report concomitant fit indices for separate models with each model containing a different criterion latent construct.

Despite the indicated limitations, the PRMS is an instrument that can be used confidently to detect positive relational management in the Italian workplace. This instrument can open new perspectives for research and intervention in organizational contexts, particularly from a positive preventive perspective (Hage et al., 2007; Kenny and Hage, 2009; Di Fabio and Kenny, 2012; Di Fabio et al., 2014, 2016a). On the basis of this framework (Hage et al., 2007; Kenny and Hage, 2009; Di Fabio and Saklofske, 2014a,b; Kenny et al., 2014; Di Fabio et al., 2016a), positive relational management needs to be promoted to enhance relational strengths also in the workplace (Blustein, 2011; Di Fabio and Kenny, 2012, 2015), improving the quality of work and lives of workers (Di Fabio and Blustein, 2016) and thereby fostering healthy organizations.

It is therefore important to create a positive workplace relational environment to enable workers to increase their personal resources (World Medical Association [WMA], 2013; Boyatzis et al., 2002, 2015; Boyatzis and Saatcioglu, 2008; Di Fabio and Bernaud, 2008; Boyatzis, 2009; Di Fabio and Maree, 2012; Rehfuss and Di Fabio, 2012; Di Fabio, 2014a, 2016b; Giorgi et al., 2014, 2015a,b, 2016; Di Fabio and Palazzeschi, 2015b; Di Fabio and Gori, 2016a,b) so that they can enhance their well-being (Snyder et al., 2014; Di Fabio and Kenny, 2016a). This study presents new ways to conceptualize organizational relationality (Di Fabio, 2015b; Di Fabio and Gori, 2016a,b) to promote gainful employment and life (Di Fabio, 2014a, 2015b), as well as to create healthy organizations and healthy businesses.

## Author Contributions

ADF conceptualized the study, chose the theoretical framework, conceptualized the items of the new scale and realized it. ADF collected and analyzed the data, wrote the methods and results. Then ADF wrote the paper, read and revised the manuscript several times.

## Conflict of Interest Statement

The author declares that the research was conducted in the absence of any commercial or financial relationships that could be construed as a potential conflict of interest.

## Appendix

**Instructions**. Please indicate with a tick the box that best describes you from 1 D strongly disagree to 5 D strongly agree.

**Table:**

	Strongly disagree	Disagree	Neither disagree nor agree	Agree	Strongly agree
1. I have respect for the value and uniqueness of others	1	2	3	4	5
2. Others have respect for my value and my uniqueness	1	2	3	4	5
3. I have respect for my value and my uniqueness	1	2	3	4	5
4. I keep a balance between respect for others and for myself	1	2	3	4	5
5. I often take care of others	1	2	3	4	5
6. Others often take care of me	1	2	3	4	5
7. I often take care of myself	1	2	3	4	5
8. I keep a balance between taking care of others and of myself	1	2	3	4	5
9. I have good relationships with my family	1	2	3	4	5
10. I have good relationships with my friends	1	2	3	4	5
11. I have good relationships with significant others	1	2	3	4	5
12. I keep a balance in my relationships with family, friends, and significant others	1	2	3	4	5
